# CovS inactivation reduces CovR promoter binding at diverse virulence factor encoding genes in group A *Streptococcus*

**DOI:** 10.1371/journal.ppat.1010341

**Published:** 2022-02-18

**Authors:** Nicola Horstmann, Kevin S. Myers, Chau Nguyen Tran, Anthony R. Flores, Samuel A. Shelburne III

**Affiliations:** 1 Department of Infectious Diseases, Infection Control and Employee Health, The University of Texas MD Anderson Cancer Center, Houston, Texas, United States of America; 2 Great Lakes Bioenergy Research Center, University of Wisconsin-Madison, Madison, Wisconsin, United States of America; 3 Center for Antimicrobial Resistance and Microbial Genomics McGovern Medical School, University of Texas Health Science Center at Houston, Houston, Texas, United States of America; 4 Department of Pediatrics, McGovern Medical School, University of Texas Health Science Center at Houston, Houston, Texas, United States of America; 5 Department of Genomic Medicine, The University of Texas MD Anderson Cancer Center, Houston, Texas, United States of America; The University of Alabama at Birmingham, UNITED STATES

## Abstract

The control of virulence gene regulator (CovR), also called caspsule synthesis regulator (CsrR), is critical to how the major human pathogen group A *Streptococcus* fine-tunes virulence factor production. CovR phosphorylation (CovR~P) levels are determined by its cognate sensor kinase CovS, and functional abrogating mutations in CovS can occur in invasive GAS isolates leading to hypervirulence. Presently, the mechanism of CovR-DNA binding specificity is unclear, and the impact of CovS inactivation on global CovR binding has not been assessed. Thus, we performed CovR chromatin immunoprecipitation sequencing (ChIP-seq) analysis in the *emm1* strain MGAS2221 and its CovS kinase deficient derivative strain 2221-CovS-E281A. We identified that CovR bound in the promoter regions of nearly all virulence factor encoding genes in the CovR regulon. Additionally, direct CovR binding was observed for numerous genes encoding proteins involved in amino acid metabolism, but we found limited direct CovR binding to genes encoding other transcriptional regulators. The consensus sequence AATRANAAAARVABTAAA was present in the promoters of genes directly regulated by CovR, and mutations of highly conserved positions within this motif relieved CovR repression of the *hasA* and *MGAS2221_0187* promoters. Analysis of strain 2221-CovS-E281A revealed that binding of CovR at repressed, but not activated, promoters is highly dependent on CovR~P state. CovR repressed virulence factor encoding genes could be grouped dependent on how CovR~P dependent variation in DNA binding correlated with gene transcript levels. Taken together, the data show that CovR repression of virulence factor encoding genes is primarily direct in nature, involves binding to a newly-identified DNA binding motif, and is relieved by CovS inactivation. These data provide new mechanistic insights into one of the most important bacterial virulence regulators and allow for subsequent focused investigations into how CovR-DNA interaction at directly controlled promoters impacts GAS pathogenesis.

## Author summary

Tight regulation of virulence factor production is a critical, but poorly understood aspect of bacterial pathogenesis. The OmpR/PhoB family member control of virulence regulator (CovR) is the master virulence factor controller in group A *Streptococcus* (GAS), a bacterium which commonly causes a diverse array of human infections. Mutations in the cognate kinase of CovR, CovS, are commonly observed among invasive GAS isolates, but the functional impact of CovS on global CovR function is unknown. Herein, we defined CovR global DNA binding locations, identified a consensus CovR binding motif, and determined how inactivation of the CovR cognate sensor kinase, CovS, impacts CovR-DNA interaction. Our findings show that CovR-repressed virulence factor encoding genes are directly regulated by CovR and that CovS inactivation markedly reduces CovR binding at CovR-repressed promoters. Given the widespread nature of CovR homologues in streptococci and other Gram-positive pathogens, these findings extend understanding of mechanisms by which OmpR/PhoB family members impact the ability of bacteria to cause serious infections.

## Introduction

The capacity of bacteria to cause infection is closely linked to their ability to modulate gene expression in response to environmental stimuli. Group A *Streptococcus* (GAS), also called *Streptococcus pyogenes*, is a major human pathogen that causes a broad array of human diseases ranging from uncomplicated pharyngitis and impetigo to life-threatening infections such as necrotizing fasciitis and streptococcal toxic shock syndrome [1]. Consequently, the ability of GAS to adapt to changing environmental conditions is fundamental to its pathogenicity and is facilitated by the coordinated expression of a large array of virulence factors.

Model organisms such as *Escherichia coli* and *Bacillus subtilis* use alternative sigma factors as a major mechanism of controlling gene expression [2]. In contrast, the role of alternative sigma factors in GAS is minimal [3], such that GAS mainly relies on the function of stand-alone regulators and two-component gene regulatory systems (TCS) to differentially impact gene transcription [4]. Although there are thirteen conserved TCS in GAS, the control of virulence (CovRS) system, also known as capsule synthesis regulator (CsrRS), has long been recognized as the most important to GAS pathogenesis given its impact on a diverse array of critical virulence factor encoding genes [5,6]. The CovRS system primarily represses virulence factor encoding gene expression, and naturally occurring mutations in CovRS result in hyper-virulent strains due to heightened production of the GAS hyaluronic acid capsule, actively secreted toxins, and cell-surface proteases [7–10]. In addition to its role in GAS pathogenesis, CovR is highly conserved amongst β-hemolytic *streptococci*, and CovR homologues are present in a wide range of important human pathogens, such as *Staphylococcus aureus* [11–13].

Despite being extensively studied for 20+ years, many questions regarding the CovRS system remain unanswered. Only recently has a global analysis of CovR binding *in vivo* been published [14]. In that study, the authors performed chromatin immunoprecipitation and DNA sequencing (ChIP-seq) on GAS grown either in the presence of LL-37 or Mg^2+^, which decreases and increases CovR phosphorylation, respectively. Direct targets of CovR regulation were identified, including multiple regulatory proteins thought to contribute to the CovRS regulon. Importantly, no CovR binding motif was identified. Additionally, the impact of CovS inactivation, which naturally occurs in a significant percentage of invasive GAS isolates, on global CovR binding has not been determined.

In general, CovR is thought to interact with stretches of AT-rich DNA regions as they are often found in GAS promoters. To date, three similar CovR DNA binding motifs, namely ATTARA (with R being either A or G, [15]), TATTTTAAT (CovR motif from group B *Streptococcus*, [12]), and TWATTTTTAAWAAAAM (with W being A or T, and M being A or C, [16]) have been proposed, of which the ATTARA motif has been most frequently used to identify potential binding sites in CovR-regulated promoters [6]. However, albeit found in many CovR-regulated promoters, the ATTARA motif (just as the other two motifs) has its shortcomings in terms of explaining CovR DNA binding properties and CovR-mediated gene regulation mechanisms. With more than 4000 ATTARA sequences in the GAS genome [17], the number of potential binding sites greatly surpasses the number of genes regulated by CovR as well as the number of estimated CovR molecules in the cell [6,15]. Mutation of some ATTARA sites in well-known CovR-regulated promoters did not impact gene regulation [18,19], while in turn CovR has been shown to bind to other AT-rich DNA regions devoid of the ATTARA recognition sequence [20,21]. Importantly, none of the current motifs align with the commonly accepted paradigm of DNA binding characteristics employed by OmpR/PhoB family proteins [22–27]. Typical for a member of the OmpR/PhoB family of transcriptional regulators, phosphorylation of CovR on a conserved aspartate residue stabilizes homodimerization of the protein thereby increasing its DNA affinity [17]. Several CovR homologs have been shown to interact with two tandem binding sites separated by four to six base pairs [22–27] which allows binding of the two monomers on the same side of the DNA. Accordingly, phosphorylated CovR (CovR~P) is also suspected to bind as dimer to tandem sites in either head-to-head or head-to-tail orientation [6], yet paradoxically the current CovR binding motifs are normally not found in tandem with spacing suitable for dimer binding.

Thus, to enhance insight into mechanisms underlying CovR DNA binding characteristics, we herein globally determined CovR DNA binding sites in the *emm1*-type GAS strain MGAS2221 and used these data to generate a DNA binding motif. Additionally, we assessed the impact of CovS inactivation on CovR DNA binding. Our findings show that CovR directly binds to the promoter region of a broad array of GAS virulence factor encoding genes and that CovS inactivation decreases CovR promoter binding at CovR-repressed virulence factor encoding genes.

## Results

### Chromatin immunoprecipitation (ChIP) enriches DNA from the CovR regulated promoters of *ska* and *sagA*

**Fig 1:**
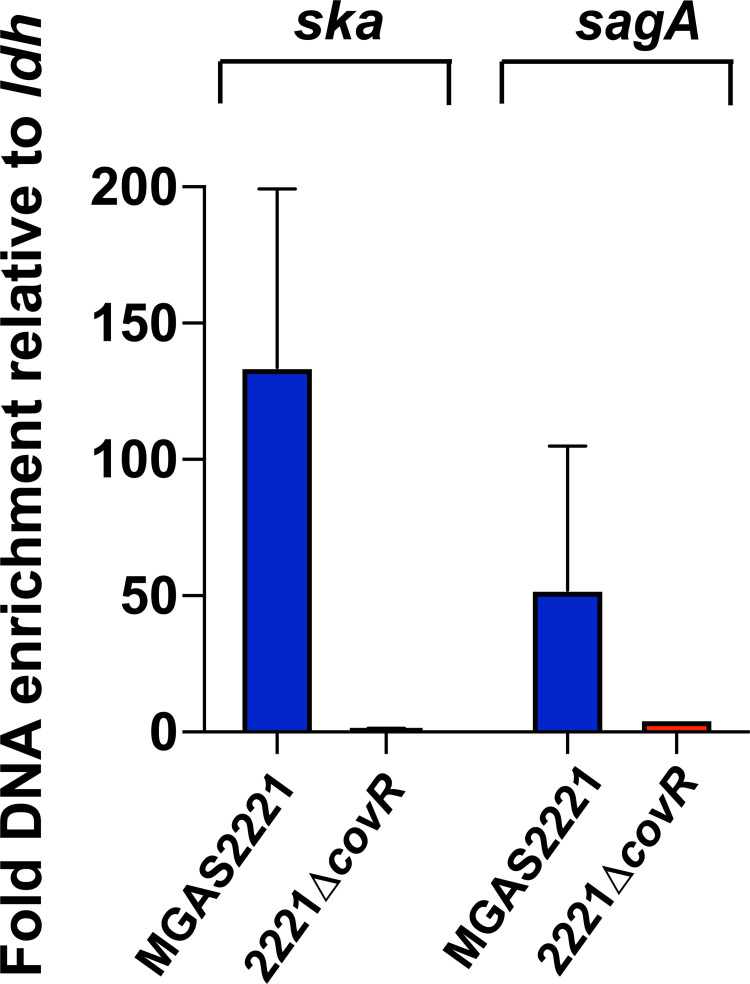
ChIP using anti-CovR antibody enriches *ska* and *sagA* promoter DNA. SYBR qPCR analysis to quantify enrichment of *ska* and *sagA* promoter DNA in ChIP samples obtained from GAS strains MGAS2221 and 2221Δ*covR* using anti-CovR_ND_ antibody. Values were normalized to fold-enrichment of the *ldh* promoter, a gene whose expression is not affected by CovR. Measurements were done in duplicate with four biological replicates grown in THY to mid-exponential phase. Data graphed are mean ± standard deviation.

To determine CovR DNA binding sites in GAS, we first sought to prove our ability to enrich CovR-bound DNA sequences by chromatin immunoprecipitation (ChIP). To this end, the *emm1* strains MGAS2221 (wild type) and 2221Δ*covR* (control) (Table 1) were grown to mid-exponential phase in THY medium. CovR-bound sheared DNA was immunoprecipitated using an antibody directed against the N-terminal domain of CovR (anti-CovR_ND_). To assess the quality of the obtained ChIP DNA samples, CovR-specific enrichment of *ska* (encoding streptokinase) and *sagA* (first gene of the *sag* operon encoding streptolysin S) promoter region was quantified by SYBR qPCR (Fig 1). CovR controls the expression of *ska* and the *sag* operon, and *in vitro* binding of recombinant CovR to these promoters has been shown previously by DNA footprint experiments [18,21]. Consistent with *in vivo* CovR binding, we detected significant enrichment of *ska* promoter DNA in the wild type ChIP samples compared to input DNA. Similarly, we observed CovR-specific enrichment of *sagA* promoter DNA, although fold-enrichment was less pronounced compared to the *ska* promoter (Fig 1). No enrichment of either promoter DNA was observed in 2221Δ*covR*, indicating specificity of our anti-CovR_ND_ antibody (Fig 1).

**Table 1:** Strains and plasmids.

Strains	Description	
MGAS2221	Clinical isolate, *emm1*, *covRS* wild type	[28]
2221Δ*covR*	MGAS2221, Δ*covR*:*aphA3*	[29]
2221-CovS-E281A	MGAS2221, exchange of glutamate at CovS position 281 to alanine	[30]
2221-CovR-D53A	MGAS2221, exchange of aspartate at CovR position 53 to alanine	[30]
*E*. *coli* DH5α	*Sup*E44,Δ*lac*U109,(Φ80*lac*ZΔM15), *hsd*R17, r*ec*A1,*end*A1,*gqr*A96, *thi*-1,*rel*A1	[31]
Rosetta (DE3)/pLysS		Novagen
**Plasmids**		
pET15b	Overexpression vector, N-terminal His_6_-tag, *amp*^*R*^	Novagen
pET15b-CovR_ND_	Overexpression of CovR, N-terminal domain	this study
pJC306	*luxAB* transcriptional fusion, *spec*	gift from M. Federle
pJC306-*hasA*	*hasA*-*luxAB* transcriptional fusion	this study
pJC306-*hasA*_mut_	*hasA*-*luxAB* transcriptional fusion with mutation in putative CovR binding motif	this study
pJC306-*spy_0187*	*spy_0187*-*luxAB* transcriptional fusion	this study
pJC306-*spy_0187*_mut_	*spy_0187*-*luxAB* transcriptional fusion with mutation in putative CovR binding motif	this study
pJC306- *spy_0187*_sp_	*spy_0187*-*luxAB* transcriptional fusion, with 2bp insertion within putative CovR binding motif	this study

### Identification of CovR DNA binding sites in MGAS2221 genome

**Fig 2:**
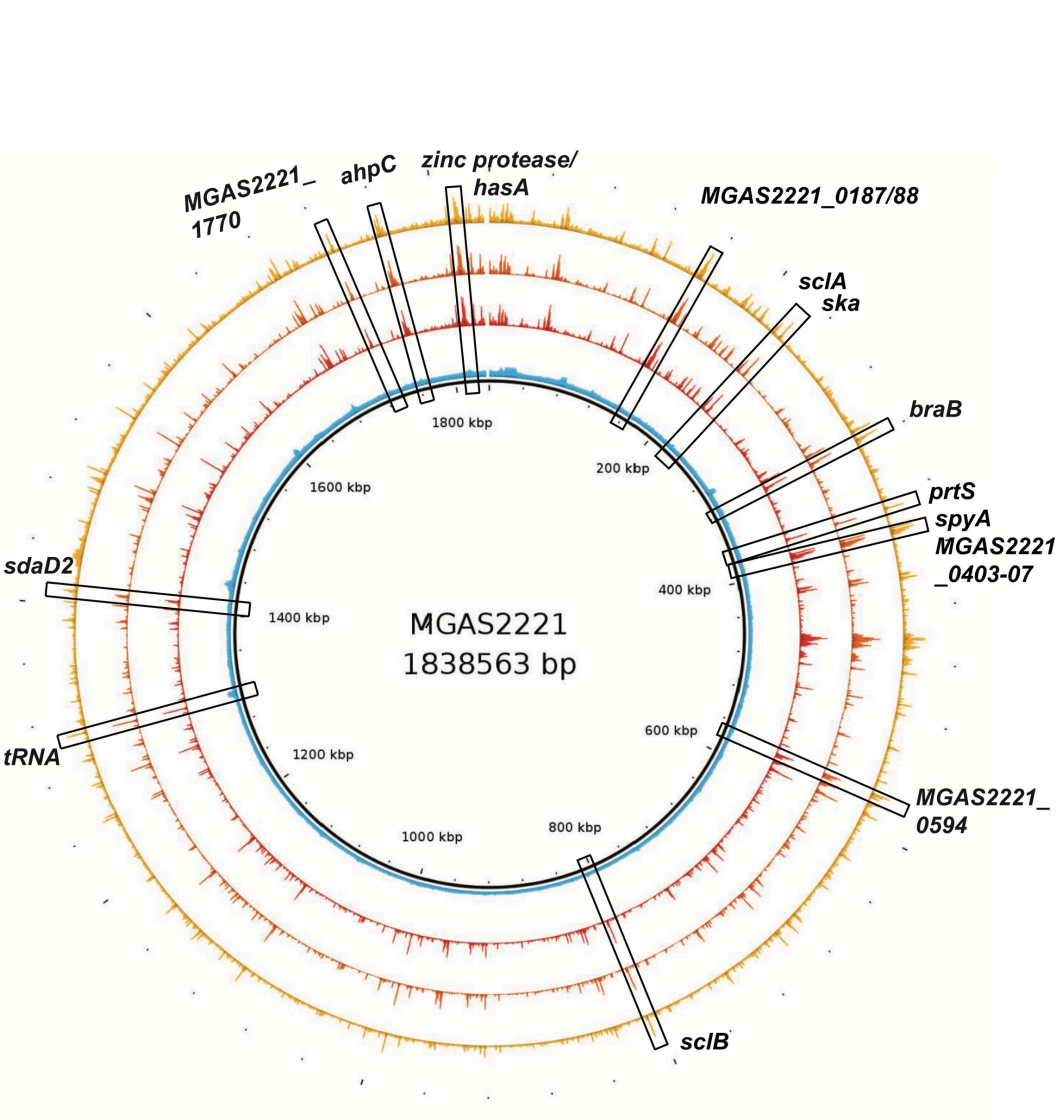
Genomic location of CovR binding sites. Map of the MGAS2221 genome with distribution of sequencing reads obtained from ChIP-seq. Sequencing reads of three representative samples of MGAS2221 (shown in yellow, orange, and red) and one 2221Δ*covR* control sample (shown in blue) are depicted. Representative peaks (enriched sequencing reads) at strongly enriched promoters (RPKL >500) are highlighted in boxes and labeled. Gene numbers refer to open reading frames from the MGAS2221 genome (NZ_CP043530.1). Note, that strain MGAS2221 has a chromosomal inversion relative to strain MGAS5005 resulting in altered location of *ska*.

To globally assess CovR DNA binding, four independent ChIP-enriched DNA samples per strain were subjected to massively parallel DNA sequencing. The generated reads of each ChIP-seq dataset were aligned to the MGAS2221 genome. Fig 2 depicts the reads obtained from MGAS2221 and 2221Δ*covR* samples plotted against the genomic location. The data reveal excellent reproducibility and a low background. As anticipated from our earlier qPCR-based assessment, a strong peak in the *ska* promoter was detected in all MGAS2221 samples (Fig 2). In contrast, no significant enrichment was observed in the ChIP-seq datasets of control strain 2221Δ*covR* in this region (Fig 2), further emphasizing the specificity of our anti-CovR antibody.

Using significant enrichment parameters as defined in the Materials and Methods, we identified 74 CovR binding sites in the MGAS2221 genome (Tables 2 and 3). Of these, 42 (57%) were associated with genes that have been previously established as part of the CovR regulon by diverse transcriptome analyses and *in vitro* data [30,21,20] (Table 2), whereas 32 peaks were in proximity to genes not known as being CovR regulated (Table 3). A large majority of the binding sites (61/74, 82%) were located within promoter regions, whereas only 13 peaks were located within a gene coding region. Thus, CovR DNA binding is strongly biased toward non-coding promoter DNA as expected for a transcriptional regulator. Similarly, of the 42 DNA binding sites which were in proximity to known CovR-regulated genes, 39 (93%) were in promoter regions. Moreover, using reads per kilobase length (RPKL), a normalized metric of amount of precipitated DNA, we found that enrichment was usually stronger at promoter DNA compared to intergenic regions (average RPKL value of 472 vs. 235). Likewise, the average RPKL value for peaks at known CovR-regulated genes was twice as high compared to non-regulated genes (585 vs. 280).

**Table 2:** Peaks at genes belonging to CovR regulon.

M5005 Spy#	MGAS2221 #	Gene	location	CovR binding in MGAS2221	CovR binding in 2221-CovS-E281A	RPKL ratio MGAS2221/2221-CovS-E281A	Transcript level ratio: 2221-CovS-E281A/MGAS2221
**Virulence factor encoding genes**							
*_0139*	*_0183*	*nga*	promoter*	**+**	-	7.49	4.98
*_1687*	*_0251*	*sclA*	promoter	**+**	**+**	4.73	38.63
*_1684*	*_0254*	*ska*	promoter	**+**	**+**	2.65	1.53
*_0281*	*_0340*	*dahA*	promoter	**+**	**-**	2.69	1.78
*_0341*	*_0397*	*prtS*	promoter	**+**	**+**	3.57	23.81
*_0351/2*	*_0403/4*	*spyA/hypo*	promoter	**+**	**-**	6.63	4.51
*_0356*	*_0408*	*speJ*	promoter	**+**	**-**	5.39	3.80
*_0562*	*_0598*	*sagA*	promoter	**+**	**+**	1.11	0.69
*_0570*	*_0606*	*sagI*	intergenic	**+**	**-**	3.67	0.69
*_0668*	*_0700*	*mac-1*	promoter	**+**	**+**	2.59	13.80
*_0777*	*_0802*	*sclB*	promoter	**+**	**+**	3.44	1.83
*_0981*	*_0993*	*cfa*	promoter	**+**	**+**	1.23	0.31
*_0996*	*_1009*	*speA2*	promoter	**+**	-	3.14	7.66
*_1106*	*_1119*	*grab*	promoter	**+**	**+**	1.10	0.09
*_1169*	*_1179*	*spd3*	promoter	**+**	**-**	3.80	0.86
*_1407*	*_1422*	esterase	promoter	**+**	-	2.49	7.45
*_1415*	*_1430*	*sda1*	promoter	**+**	**+**	2.17	2.54
*_1715*	*_1697*	*scpA*	promoter	**+**	-	8.33	2.06
*_1718*	*_1699*	*sic*	promoter	**+**	-	8.54	2.36
*_0668*	*_1700*	*emm*	intragenic	**-**	**+**	0.36	1.14
*_1851*	*_1825*	*hasA*	promoter	**+**	**+**	6.33	17.83
**Transcriptional regulators**							
*_0034*	*_0066*	*rgg4/comX*	promoter	**+**	**+**	1.44	1.92
*_0282*	*_0341*	*covR*	promoter	**+**	**+**	0.69	0.99
*_1307*	*_1314*	*trxTRS*	promoter	**+**	**+**	0.48	0.64
*_1578/9*	*_1588/9*	XRE family protein	promoter	**+**	-	4.01	1.09/1.51
*_0195*	*_1690*	MarR family	promoter	**-**	**+**	0.61	0.32
*_0670*	*_1701*	*mga*	promoter	**+**	**+**	2.77	1.07
**Transport and metabolism**							
*_0146*	*_0189*	*metB*	promoter	**+**	**-**	4.09	0.12
*_1708*	*_0232*	*dppE*	intragenic	**+**	**+**	1.77	1.44
*_1704*	*_0236*	*dppA*	promoter	**+**	**+**	0.97	1.42
*_0274*	*_0333*	*braB*	promoter	**+**	**+**	0.88	0.59
*_0473/4*	*_0516/7*	*msf/licT*	promoter	**+**	**+**	0.59	0.35
*_1601*	*_1610*	*hflc*	promoter	**+**	-	7.77	1.30
*_0249/8*	*_1638/9*	*oppA/dacA*	promoter	**+**	**+**	2.02	1.10/1.52
*_1850*	*_1824*	zinc protease	intragenic	**+**	**+**	3.94	1.37
**Synthesis, DNA repair**							
*_1291*	*_1299*	*cas3*	promoter	**+**	-	4.09	4.15
*_1556/7*	*_1567/8*	*hypo/mutY*	promoter	**+**	-	4.15	3.38/1.73
**Unknown function/hypothetical genes**							
*_0115*	*_0159*	*hypo*	promoter	**+**	**+**	2.46	14.15
*_0142*	*_0186*	*hypo*	promoter	**+**	-	5.67	5.86
*_0143*	*_0187*	*hypo*	promoter	**+**	**+**	2.99	2.64
*_0144*	*_0188*	*hypo*	promoter	**+**	**+**	3.17	6.51
*_0354*	*_0406*	*hypo*	promoter	**+**	**+**	3.83	4.68
*_0355*	*_0407*	*hypo*	promoter	**+**	**+**	3.09	11.41
*_1209*	*_1218*	*hypo*	promoter	-	**+**	0.56	0.41
*_1731*	*_1711*	*grm/hypo*	promoter	**+**	-	4.92	1.84

* defined as peak enrichment being located within 300 bps of transcriptional start site as determined in strain S119 [32]

# transcript levels of 2221-CovS-E281A and MGAS2221 were taken from [30].

Significant peaks as defined in Materials and Methods

**Table 3:** Peaks at genes not regulated by CovR.

M5005 _Spy #	MGAS2221 #	Gene	Location	CovR binding in MGAS2221	CovR binding in 2221-CovS-E281A	RPKL ratio in MGAS2221/2221-CovS-E281A
**Virulence factor encoding genes**						
*_0182*	*_0222*	*speG*	intragenic	-	**+**	0.50
*_1531*	*_1545*	*isp2*	promoter	**+**	**-**	1.20
*_1540*	*_1552*	*endoS*	promoter	**+**	**-**	4.36
**Transcriptional regulators**						
*_0106*	*_0149*	*rofA*	intragenic	**+**	**+**	0.46
*_0805*	*_0827*	*srtK*	intragenic	**+**	**-**	3.46
*_1512*	*_1527*	*codY*	promoter	**+**	**+**	3.34
**Transport and metabolism**						
*_T0041*	*_0012*	*hpt*	intragenic	**+**	**+**	0.40
*_T0045*	*_0013*	metalloprotease	promoter	**+**	-	3.81
*_0148*	*_0191*	*sgaT*	promoter	**+**	**+**	0.69
*_0443*	*_0487*	*aroE*	intragenic	**+**	**+**	0.84
*_0444*	*_0488*	hydrogenase	promoter	**+**	**+**	1.31
*_0444*	*_0488*	hydrogenase	intragenic	**+**	**-**	1.31
*_0449*	*_0492*	dehydrogenase	promoter	**-**	**+**	0.55
*_0500/1*	*_0538/9*	riboflavin	promoter	**+**	**+**	1.39
*_0657*	*__0691*	*trxB*	intragenic	**+**	**+**	0.89
*_0713*	*_0742*	*bcaT*	promoter	**+**	**+**	0.59
*_0760/1*	*_0787/8*	Amino acid ligase	promoter	**-**	**+**	0.50
*_0829*	*_0849*	*potD*	intragenic	**-**	**+**	0.54
*_0943*	*_0958*	*cdd*	intragenic	**+**	-	3.44
*_1267*	*__1275*	*coaD*	promoter	**+**	**+**	0.51
*_1479*	*_1496*	*manL*	promoter	-	**+**	0.70
*_1501*	*_1518*	muramidase	intragenic	-	**+**	0.46
*_1513*	*_1528*	aminotransferase	promoter	-	**+**	0.47
*_1518*	*_1533*	ABC transporter	promoter	**+**	-	2.92
*_0240*	*_1646*		intragenic	**-**	**+**	0.35
*_1768*	*_1743*	*ahpC*	promoter	**+**	**+**	1.00
*_1843*	*_1817*	transglycosylase	promoter	**+**	**+**	2.99
*_1856*	*_1830/1*	*guaA/*	promoter	**+**	**+**	0.80
*_1862*	*_1835*	ABC transporter	promoter	**+**	**+**	2.42
**Synthesis, replication and cell division**						
*_0001*	*_0001*	*dnaA*	intragenic	**+**	-	1.45
*_0035*	*_0068*	*ruvB*	promoter	**+**	**+**	1.67
*_0083*	*_0128*	*rpoB*	promoter	**+**	**+**	0.80
*_0308*	*_0365*	*scp1/scpA*	intragenic	**-**	**+**	0.40
*_0506*	*_0544*	*ftsW*	promoter	**-**	**+**	0.41
	*_0594*	transposase	promoter	**+**	**+**	0.48
*_0597*	*_0633*	*ribosomal*	intragenic	**-**	**+**	0.36
*_1139*	*_1152*	*queA*	intragenic	**-**	**+**	0.41
*_1321*	*_1327*	*tRNA*	intragenic	**+**	**+**	4.16
*_1327*	*_1341*	*comfA*	intragenic	**-**	**+**	0.41
*_0239*	*_1647*	*mecA*	intragenic	**-**	**+**	0.38
*_1858*	*_1832*	*trsA*	intragenic	**+**	**+**	0.43
**Unknown function/hypothetical genes**						
*_0032*	*_0064*		intragenic	**-**	**+**	0.94
*_0032*	*_0064*		intragenic	**-**	**+**	0.49
*_0180*	*_0221*	*hypo*	promoter	**+**	-	1.62
*_0403*	*_0450*	*hypo*	promoter	**+**	**-**	3.56
*_0446*	*_0490*	*hypo*	intragenic	**-**	**+**	0.39
*_0911*	*_0927/8*	*hypo/stk*	promoter	**+**	-	2.62
*_1048*	*_1062*	*hypo*	intragenic	**-**	**+**	0.52
*_1390/1*	*_1405/6*	*hypo*	promoter	**-**	**+**	0.35
*_1794*	*_1769*	*hypo*	promoter	**+**	**+**	0.47
*_1795*	*_1771*	membrane protein	promoter	**+**	**+**	5.34

### Global correlation of CovR DNA binding with CovR transcriptome data reveal differences between CovR-repressed and -activated genes

**Fig 3:**
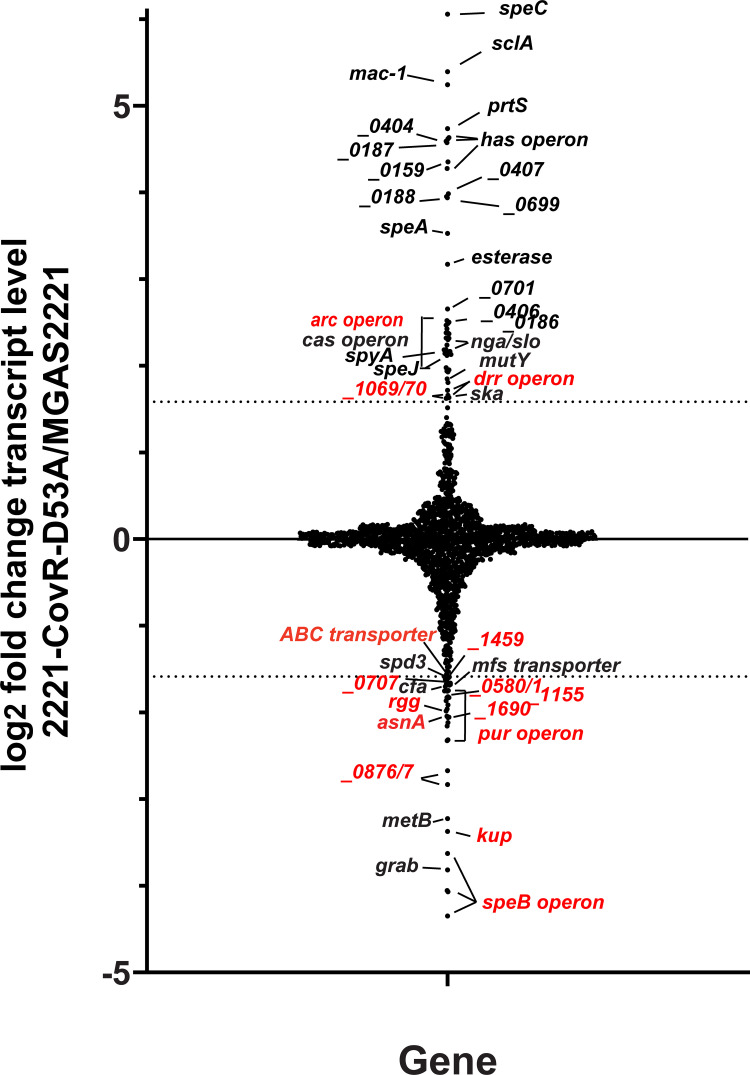
CovR directly regulates the vast majority of virulence factor encoding genes in the CovR regulon. Graphical representation of variation in gene transcript levels in 2221-CovR-D53A (no CovR~P) relative to wild type strain MGAS2221 using RNAseq data from [30]. Gene names or *spy* numbers (from the MGAS2221 genome) are provided for those genes with ≥3-fold change in transcript levels indicated by dashed lines and are labeled in black (CovR binding detected in our ChIP-seq data) or red (no CovR binding detected), respectively. Note, that for repressed genes in particular, nearly all highly impacted genes have CovR binding sites.

Alteration in CovR~P levels is currently considered the key mechanism by which the CovRS system impacts gene expression [30,33,14]. Therefore, in order to determine the degree of direct regulation within the CovR regulon, we compared our DNA binding data with previously published transcriptome data in *emm1*-type GAS, in which we correlated alteration in CovR~P levels with GAS global gene expression [30]. There were 80 genes with ≥ 3-fold change in transcript level between 2221-CovR-D53A (expressing unphosphorylated CovR) and MGAS2221 [30]. Of these, 46 had higher transcript levels (CovR~P repressed genes) and 34 genes had lower transcript levels (CovR~P activated genes) in strain 2221-CovR-D53A compared to wild type. Given that differentially expressed genes may be part of operons, there were 40 promoter regions (23 repressed and 17 activated), and hence 40 expected CovR binding sites for these 80 genes. A comparison with ChIP-seq CovR binding loci (Fig 3, labeled in black) showed a clear distinction between CovR~P repressed and CovR~P activated genes. Whereas CovR binding was identified in the promoter regions of 20 out of 23 (87%) CovR~P repressed genes/operons, only five out of 17 (29%) activated genes/operons, including the GAS virulence factor encoding genes *cfa*, *grab*, and *spd3*, had CovR binding sites (Fig 3). This finding implies that CovR~P mediated repression occurs predominantly by direct mechanisms, while CovR~P activation mechanisms are likely more complex.

### CovR directly binds promoter regions of regulated virulence factor encoding genes

**Fig 4:**
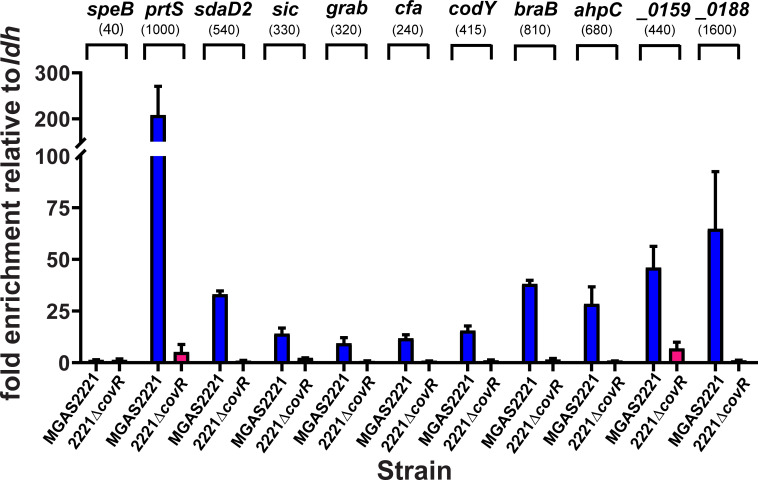
Validation of ChIP-seq binding data using SYBR qPCR. Graph depicts normalized enrichment of selected promoter DNA (labeled on top of graph) in ChIP samples obtained from denoted GAS strains as measured by SYBR qPCR. Measurements were done in duplicate with at least two biological replicates grown to mid-exponential phase with data shown being mean ± standard deviation. Given the lack of peaks in our ChIP-seq datasets in the *speB* region, SYBR primers for the *speB* promoter were chosen based on [14]. The numbers in brackets indicate the average RPKL in ChIP-seq experiment for comparison.

CovR impacts the expression of the majority of known GAS virulence factor encoding genes [34,35,10]. Thus, we next sought to determine whether our observed CovR binding sites were located in the promoter regions of GAS virulence factor encoding genes. Our ChIP-seq data identified CovR DNA enrichment in the promoter regions of *hasA* (first gene of capsule operon), *prtS* (also known as *spyCEP*, streptococcal cell envelope protease), *ska*, and *sagA*, for all of which CovR interaction has been previously established *in vitro* [15,18,21,29,36]. In fact, some of the strongest enrichment within the MGAS2221 genome was observed in the *hasA*, *prtS*, and *ska* promoter regions (Fig 2). In addition to binding at the *sagA* promoter, we detected strong DNA enrichment at the end of the *sag* operon near the end of the *sagI* gene (S1A Fig), although the functional implications thereof on gene regulation need to be evaluated. Moreover, CovR binding was observed in the promoter region of additional 17 well-known GAS virulence factor encoding genes (Tables 2 and 3). For example, CovR bound adjacent to genes coding for secreted extracellular hydrolase enzymes such as *scpA* (C5 peptidase), *nga* (NAD^+^-glycohydrolase), *endoS* (endoglycosidase), *MGAS2221_1422* encoding a secreted esterase, *spd3*, and *sda1* (DNases). We also observed enrichment in the promoter of the exotoxin encoding genes *spyA* (ADP-ribosyltransferase), *speJ*, and *speA* (superantigens). *SpeA* expression is strongly repressed by CovR, and high-level SpeA production has previously been used as marker for decreased CovR activity [29]. Likewise, we detected significant enrichment in the promoter of genes encoding the immunomodulating surface proteins SclA, SclB, IgG protease Mac-1, and GRAB (protein G-related α2-macroglobulin-binding protein). Further, there were peaks upstream of *cfa* (CAMP-factor), *sic* (streptococcal inhibitor of complement) and *dahA*, a newly identified virulence gene upstream of *covR* with so far unknown function [37]. Unexpectedly, however, we did not observe CovR binding in the promoter of the *speB* operon, which encodes a key cysteine proteinase, in our experimental conditions, which was confirmed by the lack of enrichment of *speB* promoter DNA during SYBR qPCR (Fig 4). Therefore, with the exception of *speB*, we identified CovR binding sites in the promoters of all CovR regulated virulence factors encoding genes indicating that CovR regulates virulence factor production predominantly by direct binding to promoter DNA.

### Limited identification of CovR promoter binding to transcriptional regulators in the CovR regulon

Although our data revealed that CovR primarily exerts a direct influence on virulence gene expression, CovR-mediated regulation and *in vitro* CovR interaction with promoter DNA of virulence-associated transcriptional regulators has also been reported [4,20,38,39]. Although CovR autoregulation is controversial [20,30,40], we observed enrichment of *covR* promoter DNA, albeit the extent of DNA enrichment was relatively low (average RPKL = 238) in comparison to that observed for numerous virulence factor encoding genes such as *hasA* or *prtS* (average RPKL of 1272 and 999, respectively). In contrast, unexpectedly, there was no significant enrichment identified upstream of either *rivR* or *ropB*, the latter encoding a critical *speB* regulator [41–43]. Similarly, we did not detect significant DNA enrichment adjacent to *MGAS2221_1690* (*spyM5005_0195*), a MarR transcriptional regulator and part of the RscA operon [39]. Finn et al. had recently attributed a substantial contribution of CovR indirect regulatory effects to RivR, *spyM5005_0195*, and RopB [14]. Indeed, CovR binding loci were identified in the promoter of only a limited number of transcriptional regulators belonging to the CovR regulon. Specifically, we detected CovR binding upstream of *rgg4*, which encodes a regulator for the competence-specific sigma factor ComX, and in the non-coding region between *MGAS2221_1588* and *_1589*, encoding two divergently transcribed transcriptional regulators of unknown function. In addition, we identified significant peaks upstream of the *trxTRS* operon. CovR~P positively influences the transcript levels of the *trxTSR* operon that includes genes encoding the quorum sensing-like TCS TrxSR, which has previously been identified as important for impacting GAS virulence and responding to asparagine released by host cells during infection [44,45].

In contrast, we detected CovR binding in the vicinity of several genes encoding key GAS regulators that are not part of the CovR regulon, and the functional relevance of CovR binding at these genes is therefore currently unclear. For example, we identified CovR interaction within the coding region of the *rofA* gene, which encodes a RALP transcriptional repressor that controls the divergently expressed gene *cpa* and other virulence-associated genes (S1B Fig) [46,47]. ChIP-seq further revealed CovR binding sites in the promoters of the *mga* and *codY* genes. The multi-gene activator (Mga) protein positively impacts the expression of numerous virulence genes in the early stage of infection [48,49]. However, despite strong peaks indicating CovR binding in all MGAS2221 datasets, *mga* transcript levels are repressed by CovR only at elevated CovR~P levels in this serotype as seen in the CovS phosphatase deficient mutant strain 2221-CovS-T284A [30]. Similarly, expression of *codY*, which encodes a global regulator with roles in GAS virulence and adaptation to nutritional limitation, has not been previously identified as being impacted by CovRS in transcriptome analyses [10,29,30,35]. Using TaqMan qRT-PCR, we confirmed that *codY* transcript levels are indeed not influenced by CovR at the experimental conditions used in our ChIP-seq experiment (S2 Fig). Taken together, our data identified CovR-mediated DNA enrichment upstream of a limited number of genes coding for GAS transcriptional regulators, although not all have been identified as part of the CovR regulon.

### CovR binds to DNA upstream of genes involved in transport and metabolism

In addition to virulence factors, the CovR regulon also includes a broad array of genes involved in metabolic functions [10,30,35]. In accord with these previous observations [19], we detected CovR binding in the promoter regions of the *opp* and *dpp* operons, which code for oligo- and di-peptide transport systems, respectively. CovR binding to the *dpp* promoter has previously been established *in vitro* [19]. The relevance of the additional binding site that we detected at the end of the *dpp* operon (within gene *dppE*; S1C Fig) on *dpp* regulation needs further evaluation. CovR also bound upstream of the genes *braB* and *bcaT*, which are involved in branched-chain amino acid transport and metabolism. Interestingly, expression of these genes is controlled by CodY [50]. Given our identification of CovR binding to the *codY* promoter, this may indicate a direct interconnection between the regulons of these two key transcriptional regulators, which have previously been noted to have opposing effects on the transcript levels of many critical GAS genes [51]. Additionally, we observed DNA enrichment from the promoter of *metB*, a CovR-activated gene involved in cysteine and methionine metabolism, further indicating a prominent direct involvement of CovR in controlling amino acid metabolism. Moreover, CovR bound to DNA adjacent to genes *MGAS2221_1830 (guaA)* encoding a putative glucose uptake protein, an ABC transporter, as well as in between genes *MGAS2221_0516* and *MGAS2221_0517*, encoding an MFS transporter and the transcription antiterminator LicT, respectively.

### CovR binds to DNA adjacent to hypothetical proteins

Although the function of many genes in the CovR regulon is well defined, hypothetical proteins are also consistently identified in CovR transcriptome studies [10,33,30]. In concert with these observations, we identified CovR binding adjacent to numerous open reading frames coding for small, hypothetical proteins such as *MGAS2221*_*0406/7*, *MGAS2221*_*0450* and *MGAS2221*_*1769*. Of particular interest were binding sites in the promoter of *MGAS2221_0159* as well as *MGAS2221_0187* and *_0188* (S1B and S1D Fig). These genes are established as being CovR-regulated [30], but their role in GAS pathogenesis is relatively unknown. *MGAS2221_0159* is located immediately adjacent to the fibronectin-collagen-T antigen (FCT) region and encodes a putative secreted protein of unknown function. Conversely, *MGAS2221_0187* and *MGAS2221_0188* are located immediately downstream of the *nga*/*slo* operon and are transcribed in the opposite direction. Recently, Herrera *et al*. showed that in *emm3*-type GAS, *spyM3_0132*, a gene homolog to *MGAS2221_0188*, encodes a secreted signaling peptide which positively influences the transcription of *slo* (streptolysin O) by a so far unidentified mechanism [52]. Similarly, CovR binding adjacent to *MGAS2221_1711*, a small gene of unknown function also called *grm* (gene regulated by Mga) [49], was intriguing given its location downstream and transcribed in the opposite direction of the *speB* operon, which itself lacked any CovR binding sites (S1E Fig).

### SYBR qPCR confirms CovR binding to promoter DNA of selected genes

We employed SYBR qPCR to verify our ChIP-seq results for selected genes comprising each of the aforementioned categories (Fig 4). DNA of all promoters that exhibited peaks in our ChIP-seq data, were also significantly enriched by SYBR qPCR for wild type strain MGAS2221. In contrast, no relevant enrichment was observed in the *covR* deletion samples. The fold enrichment mostly mirrored the RPKL values in our ChIP-seq experiment. For example, out of the selected genes, *prtS* and *MGAS2221_0188* promoter DNA showed the highest enrichment by SYBR qPCR and had the highest RPKL values by ChIP-seq (Fig 4). Despite the lack of CovR regulation (see above), *codY* promoter DNA was also significantly (~15-fold) enriched over input DNA, thus affirming our ChIP-seq data (Fig 4).

### Analysis of ChIP-seq data identifies novel extended CovR DNA binding motif

**Fig 5:**
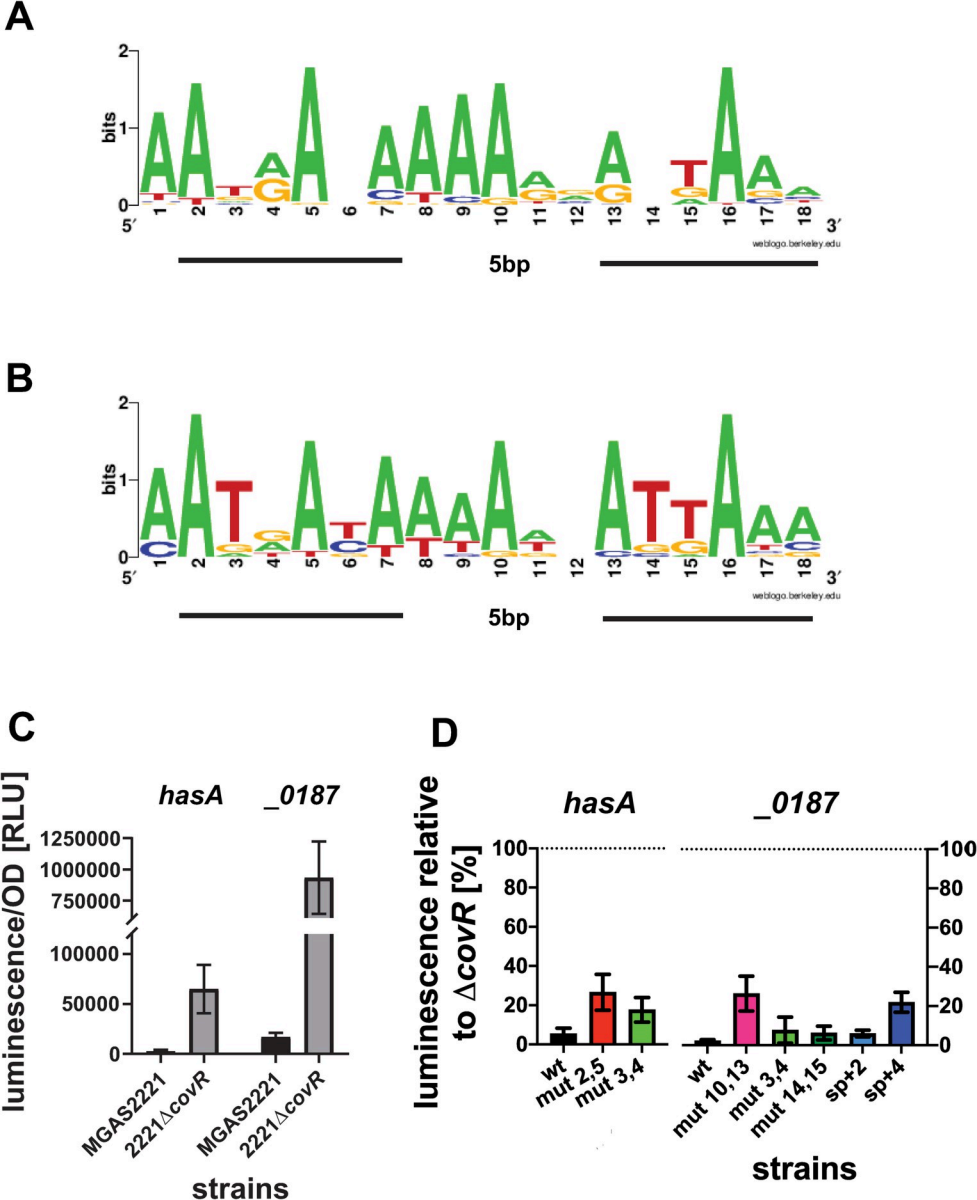
Delineation and validation of a CovR binding motif. Sequence logos generated by WebLogo [71] using CovR binding sites found by MEME generated from *(A)* 45 genes directly regulated by CovR and *(B)* the 16 genes with peaks featuring the highest reads per length (RPKL >500). The two tandem repeat motifs are underlined, respectively. *(C) luxAB* reportergene assay determining CovR repression of *hasA* and *MGAS2221_0187* in wild type strain MGAS2221 and relief of repression in strain 2221-Δ*covR* as measured in RLUs. *(D)* Effect of mutations within the putative CovR binding site in the promoters of *hasA* and *MGAS2221_0187* on gene transcription. Relative luminescence (RU per OD) is depicted in relation to luminescence in the respective 2221Δ*covR* background, which represents the unrepressed state (set to 100%). Cells of three biological replicates were grown in THY at two independent experiments with data shown being mean ± standard deviation. mut x,y: mutation of two bases to cytosine at position x and y within the putative DNA binding motif found in the promoters of *hasA* or *MGAS2221_0187*, respectively. sp+2 and sp+4: insertion of two or four guanosines in the 5bp spacer region of the putative motif.

Given the shortcomings of the current ATTARA motif to define CovR DNA binding, we next sought to utilize our ChIP-seq data to establish an improved *in vivo* CovR DNA binding sequence. To optimize our ability to identify a functional consensus motif, we omitted CovR binding sequences with RPKL values close to the threshold of 100 and whose transcript levels were not impacted by CovR. We employed the MEME suite [53,54] to search for potential CovR binding motifs among 45 regions of enrichment encompassing 400 bp around the average peak location, respectively. Using an 18-20bp motif length setting, we identified a binding logo (Fig 5A) with the consensus sequence 5’-AATRANAAAARVABTAAA-3’ (with R being A or G, N being any nucleotide, V being A, C or G, and B being C, G or T) with a statistically significant E-value of 3.5e^-10^. Interestingly, running our identified motif in TomTom (a program included in MEME suite, which compares provided motifs to transcription regulator motif databases [55,56], showed the similarity of our motif to the previously identified CovR binding sequence from group B *Streptococcus* [12], thereby increasing the confidence of our identification. The putative CovR DNA binding sequence consists of two tandem motifs of six base pairs closely resembling the previously proposed ATTARA motif separated by a five base pair long spacer, which places the two tandem motifs in a head-to-tail fashion on the same side of the DNA. This arrangement is consistent with DNA binding motifs that have been identified for other members of the OmpR/PhoB family of transcriptional regulators (S3 Fig). The DNA binding motif calculated from the CovR binding sites used in our analysis revealed strong conservation of adenosines at positions 2, 5, 7–10, and 16 (Fig 5A). Interestingly, the thymine positions in the ATTARA motif previously proposed to be crucial for CovR binding by uracil interference experiments [15], were not particularly conserved in this new DNA binding motif. However, a MEME search utilizing only the 16 sequences with the highest RPKL values (>500) from our ChIP-seq dataset yielded a highly similar motif with the consensus sequence AATRAYAAAAWBATTAAA, albeit with increased conservation of the internal TTs in the second tandem binding site (Fig 5B). Thus, the internal thymines may have an important role for high affinity CovR binding but may not be essential for CovR-DNA interaction in general.

### Mutational analysis confirms role of the newly found CovR consensus motif in CovR mediated regulation

In order to confirm the importance of the CovR binding motif for CovR-mediated transcriptional regulation, we employed a luminescence-based reporter gene assay. To this end, a transcriptional fusion of the CovR-regulated promoters *hasA* or *MGAS2221_0187* with the *luxAB* reporter gene was provided on a plasmid in the MGAS2221 and 2221Δ*covR* background, respectively, and the luminescence signal per OD was detected as a measure of promoter activity (Fig 5C). As expected, for both *hasA* and *MGAS2221_0187* wild type promoters, luminescence was virtually absent in the MGAS2221 background indicating strong repression of gene expression by CovR. This repression was relieved in the *covR* deletion background (Fig 5C). The exchange of adenosine bases at two highly conserved positions in the putative CovR binding site within the *hasA* (position 2 and 5) or *MGAS2221_0187* (position 10 and 13) promoter to cytosine, resulted in partial relief of repression of gene expression indicated by ~30% luminescence relative to the maximal expression in the respective Δ*covR* background (Fig 5D). In contrast, mutation of the two non-conserved internal TT bases within the motifs (positions 3/4 and 14/15) had only minor effect on gene regulation of the studied promoters. Further, an insertion of four base pairs in the spacer region of the putative CovR motif within the *MGAS2221_0187* promoter which would place the two tandem motifs on opposite sites of the DNA also partially relieved repression, while the introduction of only two base pairs had little influence on *luxAB* expression suggesting some flexibility in the spacer length.

Together, these results emphasize an important role of the tandem repeat motifs with suitable spacing for CovR binding and CovR-mediated transcription regulation.

### Inactivation of CovS kinase activity influences global DNA binding activity

**Fig 6:**
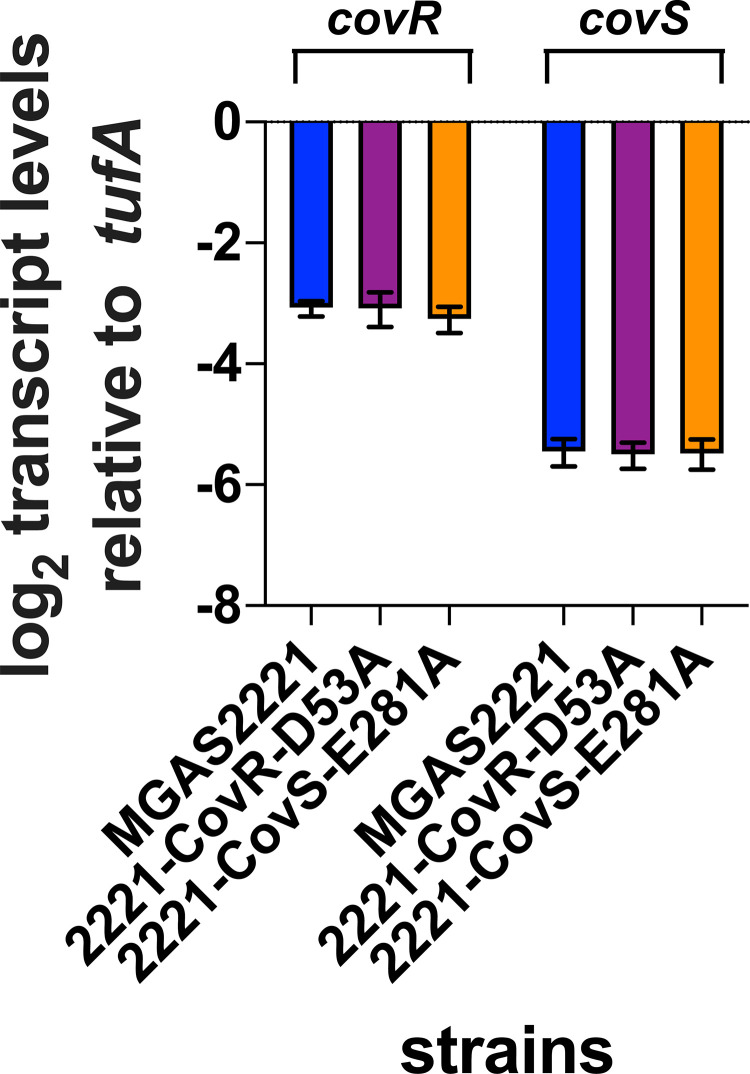
CovRS transcript levels are not affected by mutation of CovR D53 or CovS E281. *In vivo* transcript levels of *covR* and *covS* in strains MGAS2221 (blue), 2221-CovR-D53A (purple), and 2221-CovS-E281A (orange) grown to mid-exponential phase in THY medium as measured by TaqMan qRT-PCR. Data graphed are means ± standard deviation derived from four independent biological replicates measured in duplicate.

**Fig 7:**
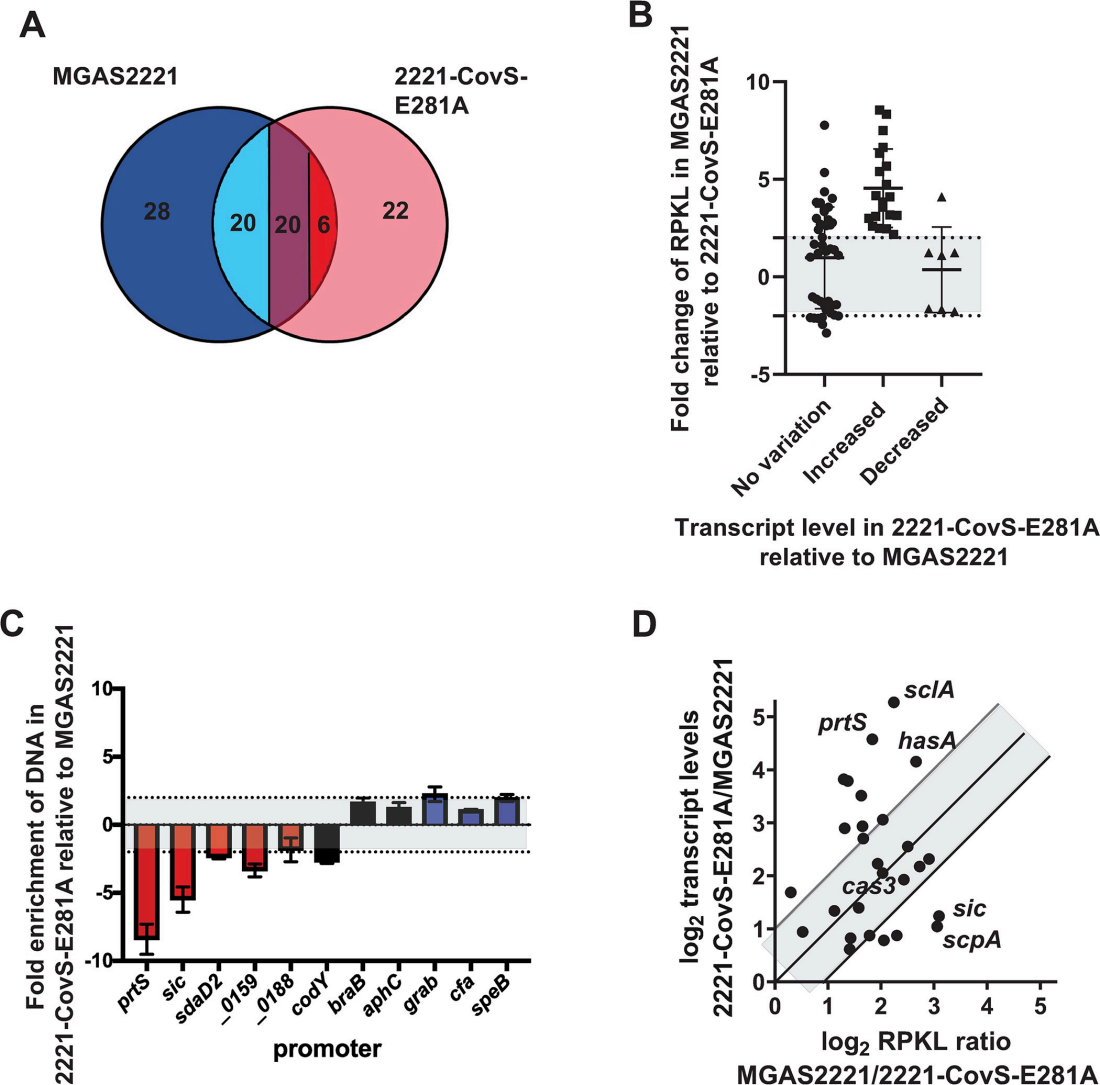
Influence of CovR phosphorylation on DNA binding. (*A*) Venn diagram depicting number of binding loci in strains MGAS2221 (blue) and 2221-CovS-E281A (pink). Note that binding sites both strains had in common can be further divided by relative RPKL ratios (>2-fold higher RPKL in MGAS2221 (light blue), no significant difference (purple), and >2-fold higher RPKL in 2221-CovS-E281A (red). (*B*) RPKL ratio of MGAS2221/2221-CovS-E281A for particular genes stratified by ≥ 2-fold variation in transcript level variation (transcript level data from [30]). Only genes with CovR binding loci in promoter regions were included. Gray area between dotted lines indicates no significant (< 2-fold) changes in RPKL ratio. (*C*) SYBR qPCR analysis showing CovR-mediated DNA enrichment of selected promoters in strain 2221-CovS-E281A relative to MGAS2221. Bars are colored in red for CovR~P repressed promoters, in black for promoters not regulated by CovR~P, and in blue for CovR~P activated promoters. Data shown are mean ± standard deviation of at least two biological samples measured in duplicate. Gray area indicates area of <2-fold differences in enrichment. *(D)* Correlation of change in transcript levels and change in RPKL values between strains MGAS2221 and 2221-CovS-E281A. Selected genes mentioned in the text are labeled. Greyed area indicates RPKL/transcript level variation ratios between 0.5 and 2 with middle line on 1.

It is well-established that spontaneous CovS inactivating mutations can arise during infection leading to hypervirulent GAS strains, and the M1T1 strain MGAS2221 readily develops such mutations during mouse infection [7–9,30,57]. Hence, to study the consequences of CovS inactivation on CovR global DNA binding, we next performed ChIP-seq using strain 2221-CovS-E281A. This strain is CovS kinase activity deficient, has CovR~P levels identical to a *covS* deletion mutant and has transcriptome data available [30]. Further, qRT-PCR analyses confirmed that the E281A exchange in CovS does not impact CovRS expression levels (Fig 6). ChIP-seq analysis revealed a total of 68 significantly enriched DNA locations, of which 46 (68%) were located within promoter DNA. However, whereas 42/74 (56%) of CovR binding sites were detected in approximation to CovR regulated genes in strain MGAS2221, only 26/68 (38%) of CovR binding sites were associated with CovR regulated genes in strain 2221-CovS-E281A (p = 0.03). Forty-six binding loci were identified in both strains, and CovR binding at 20 of these sites was markedly reduced in strain 2221-CovS-E281A (RPKL ratio MGAS2221/2221-CovS-E281A>2). In addition, 28 regions showed significant enrichment only in the wild type strain MGAS2221 (Fig 7A). Thus, CovR-mediated DNA enrichment at 48 loci is strongly dependent on high CovR~P. Importantly, this accounts for 76% (32 out of 42) of all genes directly regulated by CovR, including most virulence factors encoding genes. Moreover, 42/48 (87.5%) of CovR~P dependent binding sites were located in promoter regions (Table 2). In contrast, 22 CovR binding sites were exclusively detected in strain 2221-CovS-E281A, and six additional sites had ≥2-fold higher enrichment compared to the wild type strain (Fig 7A). Of these 28 genes, only two (*emm* and *speG*, both intergenic binding) encode known or putative virulence factors. Instead, these genes are mainly involved in metabolism, other cellular functions or are hypothetical. Moreover, 24 out of the 28 genes (86%) are not regulated by CovR, and 17 had intergenic binding loci (i.e. not promoter regions) (Tables 2 and 3). These findings suggest that CovR DNA binding exclusively or to a higher degree (i.e. higher RPKL) identified in strain 2221-CovS-E281A may not have significant functional impact.

### Elevated transcript levels of CovR-repressed genes following CovS inactivation is due to decreased CovR promoter binding

Next, we sought to determine whether varying CovR~P levels resulted in changes in CovR-mediated DNA enrichment in promoter regions that could be related to impact on gene expression. Therefore, we compared fold changes in CovR-mediated DNA enrichment (RPKL values) and transcript level variation between strains MGAS2221 and 2221-CovS-E281A. For the analysis, we utilized all genes that evidenced DNA enrichment by CovR in a promoter region in strain MGAS2221 or 2221-CovS-E281A (n = 74). The promoters were broken down into three categories contingent on their CovR~P dependent variation in transcript levels (e.g. increased, no change, or decreased transcript levels in strain 2221-CovS-E281A relative to MGAS2221; Fig 7B). Strikingly, all genes whose transcript levels were increased in strain 2221-CovS-E281A compared to MGAS2221 (CovR~P repressed genes, n = 19), had a ≥2-fold increase in RPKL values in strain MGAS2221 relative to strain 2221-CovS-E281A, indicating a CovR~P binding dependency at these loci. In contrast, for genes whose transcript levels were decreased in 2221-CovS-E281A compared to MGAS2221 (CovR~P activated genes), we observed no significant difference in RPKL values in strain 2221-CovS-E281A relative to strain MGAS2221 for eight out of nine promoters. The single exception was *metB*, whose RPKL value was markedly increased in the wild type strain compared to 2221-CovS-E281A (Fig 7B). The impact of decreasing CovR~P levels on the relative RPKL values was heterogenous for genes whose transcript levels were not influenced by CovR~P status (Fig 7B). The effect of varying CovR~P levels on promoter binding was confirmed by SYBR qPCR for selected genes comprising each category (Fig 7C).

Finally, we sought to determine whether the changes in transcript levels correlated with alterations in RPKL values in strain 2221-CovS-E281A. For this analysis, we focused on genes that were CovR~P repressed and had CovR binding in their promoter regions (n = 27, see transcript levels increased in Fig 7B). Although promoter binding was always positively influenced by CovR~P for CovR-repressed genes, we did not identify a significant correlation (Pearson correlation coefficient = 0.07, p = 0.73) between variation in RPKL values and transcript levels (Fig 7D and Table 2). While for some genes changes in transcript level were directly proportional to alteration in RPKL values (see Fig 7D; points within gray area, for example *cas3*), for other virulence factor encoding genes (e.g. *hasA*, *prtS*, and *sclA*) a small change in RPKL value was associated with a strong impact on transcript levels. In turn, despite markedly higher RPKL values at higher CovR~P levels, the transcript level of genes such as *scpA* or *sic* only showed about 2-fold variation (Fig 7D).

Taken together, we conclude that CovS inactivation markedly reshapes global CovR binding with particular impact on CovR binding affinity at the promoters of CovR-repressed virulence factor encoding genes.

## Discussion

The CovRS TCS is central to the tight regulation of a broad collection of potent virulence factors, that allow GAS to cause a variety of infectious syndromes even in previously healthy persons [5,35,58–62]. Herein, using ChIP-seq we show that CovR directly regulates at least nineteen key GAS virulence factor encoding genes, which account for nearly all virulence factor encoding genes in the CovRS regulon. Given the critical role of CovS inactivation in the development of hypervirulent GAS, we also examined the impact of CovS inactivation on global CovR binding. These data help define the direct vs. indirect CovR regulon and clarify the mechanism by which CovS inactivation results in altered production of GAS virulence factors.

While we were in the final stages of data analysis for this manuscript, the first report of a global CovR DNA binding study was published by Finn et al. [14]. Despite a few technical differences between this report and our data such as the employment of a different antibody and different strain/growth conditions that result in slightly different CovR~P levels, the overall results were quite similar, with the same CovR occupancy observed for virulence factor encoding genes, except that we did not detect CovR binding to the *speB* promoter. In addition to direct regulation by CovR, Finn *et al*. explored the indirect nature of CovR regulation by studying two genes they identified as being directly regulated by CovR that encode regulatory proteins, *spy0186* (*rivR*) and *spy0195* (regulator in the *rscA* operon; spy numbers used in Finn et al. were from strain MGAS5005). Neither of these genes were identified as having CovR enrichment in our study likely due to differences in cut-offs for calling statistically significant peaks given that most binding sites exclusively identified in Finn et al. had low (< five-fold) enrichment over mock control (e.g. *lctO*, *alaS*, *rivR* or *ffh*) [14]. Given that RivR is naturally inactivated in both *emm3* and *emm4* GAS, *emm* types in which CovS inactivation is common amongst clinical strains and markedly impacts strain virulence [63–65], and the fact that the 16 genes whose transcript levels were most increased by abrogating CovR~P [30], all are directly regulated by CovR (black genes in Fig 3), we believe that both data sets support the concept that relief of direct CovR repression induced by decreased CovR~P levels following CovS inactivation or exposure to LL-37 is the major mechanism by which CovR impacts gene expression.

Importantly, in contrast to other recent CovR ChIP-seq analyses [14,40], we were able to determine a novel CovR DNA binding motif using our ChIP-seq data by performing an extensive search in MEME. Finding a CovR DNA binding motif has long been a challenge, in part due to the extremely AT-rich sequences in the promoters of CovR regulated genes. Moreover, the proposed possible arrangement of tandem motifs in both head-to-head and head-to-toe orientation hampers motif identification [6]. Although mutations in the ATTARA sites seemed to affect CovR-mediated regulation for selected promoters (e.g. *has*, *ska*, *dpp*) [18,15,19,38], the ATTARA motif fails to explain CovR regulation globally. 5’-AATRANAAAARVABTAAA-3’ is the first CovR consensus binding sequence generated from a large quantity of sequences (45 vs. five) and comprises two tandem motifs exhibiting ATTARA similarities separated by five base pairs. It not only displayed a low E-value of 3.5e^-10^ by MEME indicating statistical significance and overlaps with binding regions previously identified in DNase footprint analyses (CB-4 in *hasA* and CB-3 in *ska* promoter) [15,18], but is also consistent with the established paradigm of DNA binding of OmpR-PhoB family proteins. Crystal structures of OmpR/PhoB family members in complex with DNA have revealed dimeric proteins bound in a head-to-tail orientation to two tandem motifs separated by a 4–6 bp spacer [22–27]. Despite the general similar composition of binding sites, the different operator sequences bound by OmpR/PhoB family proteins display distinctions in the quantity and position of specific base contacts, which are not limited to the tandem motif but also occur within the spacer (see S3 Fig).

In our CovR DNA binding motif, several adenosines were highly conserved which may indicate specific base contacts with the protein. In contrast, despite the similarity to the ATTARA motif, the two internal thymine positions, previously identified as crucial for CovR mediated regulation [15], were not particularly conserved. In order to establish general validation of our putative CovR binding sequence as crucial for CovR mediated gene regulation, we mutated positions based on conservation of bases. For both *hasA* and *MGAS2221_0187* promoters, mutation of two adenosines, respectively, partially relieved repression of gene expression (to ~30%) in a reporter gene assay. Considering that there are likely numerous other specific and unspecific interactions of CovR with the binding site and potentially more than one binding site per promoter, a complete relief of repression is not expected [15]. Our data also imply that correct spacing of the two tandem sites is important for CovR-mediated gene regulation. Further detailed biochemical and structural work will be needed to delineate the particular contribution of each base for CovR binding specificity and affinity. This in turn could allow understanding for how CovR phosphorylation directs non-specific CovR-DNA binding at AT-rich DNA regions towards a specific, regulatory functional interaction with promoter DNA.

It has long been recognized that CovS inactivation occurs in many invasive GAS strains [57,66,9]. Moreover, in animal challenge and *ex vivo* human infection models, CovS inactivation generally increases GAS virulence, particularly in *emm1* strains [10,7,8]. Importantly, CovS inactivation decreased CovR DNA binding in the promoter regions of all directly regulated virulence factor encoding genes whose transcript levels were significantly increased by CovS inactivation. This suggests that the GAS transcriptome could be quickly remodeled by CovS inactivation with a rapid increase in production of a broad array of virulence factors which would likely assist with emergence of CovS inactivated subpopulations during interaction with host immune cells. However, the impact of CovS inactivation on CovR DNA binding did not always result in similar changes in transcript levels. For example, we observed a four to six-fold decrease in CovR occupancy in the promoters of *hasA*, *prtS*, and *sclA* following CovS inactivation but a transcript level increase of 15-fold or greater. Conversely, for *sic* and *scpA*, CovS inactivation resulted in an ~8-fold decrease in CovR occupancy yet only a ~2-fold transcript level change. CovR~P is known to interact with RNA polymerase at the *hasA* promoter which causes gene expression to be highly sensitive to changes in CovR~P levels [17]. Whether similar mechanisms are at play for *prtS*, *sclA*, and other CovR-regulated genes with high transcript level changes relative to CovR occupancy variation following CovS inactivation is not currently known. Importantly, given that we did not observe a linear relationship between higher CovR occupancy and transcript level changes following CovS inactivation, simple variation in CovR promoter affinity is unlikely the sole attribute that accounts for the observed variance in transcript levels but instead underscores the complexity of gene regulation by OmpR/PhoB proteins [67].

The generation of two global analyses of CovR *in vivo* binding in GAS (this study and [14]) and another recent study in group B *Streptococcus* [40] provides important knowledge about this critical regulator, yet key questions remain regarding the role of CovR in streptococcal pathophysiology. Both we and Finn *et al*. identified CovR binding in the promoter regions of genes not regulated by CovR including the gene encoding the master metabolic regulator CodY and *ahpC* which encodes a protein involved in defense against oxidized molecules [68]. One possible explanation for this observation is that at these genes CovR promoter binding alone is not sufficient for regulation [19] but requires co-factors only present at specific circumstances, such as during infection. Another yet to be explained aspect of CovR regulation is that some of the highest CovR peaks were observed in the promoters of unstudied hypothetical proteins such as *MGAS2221_0159*, *MGAS2221_0406*, and *MGAS2221_1771* indicating that much remains to be learned about the effect of genes regulated by CovR. Similarly, the CovR binding patterns we observed suggest that the impact of CovR on gene expression is likely to be more complex than simply binding to promoter regions. For example, for genes like *covR*, *codY*, and *hasA*, we observed large peaks both in the respective promoter regions as well as 1000 bp+ upstream suggesting that CovR could impact gene expression via DNA looping or other mechanisms that involve binding sites distal from promoters. Conversely, for the *sag* and *dpp* operons we identified CovR peaks both in the promoter of the 1^st^ gene but also at the end of final gene suggesting that operon transcription could be impacted by CovR by multiple methods. Finally, for genes whose transcript levels are increased by CovS inactivation, such as *grab* and *cfa*, we did not observe a marked change in CovR promoter occupancy following CovS inactivation. Thus, it does not appear that unphosphorylated CovR has higher affinity for these promoters, and therefore the mechanism of CovR~P mediated activation remains enigmatic at present and may involve additional co-factors.

In summary, herein we provide a global analysis of CovR binding both in a wild type and CovS inactivated strain. We have used these data to generate a DNA binding motif of CovR binding. Together with other recently published results, our findings show that CovR directly regulates a vast repertoire of GAS virulence factor encoding genes and that CovS inactivation significantly reduces *in vivo* CovR DNA binding. These data markedly extend understanding of a critical aspect of the pathophysiology of a major human pathogen and provide the basis for future investigations into novel aspects of GAS pathogenesis.

## Material and methods

### Bacterial strains, media and growth conditions

Bacterial strains and plasmids used in this study are listed in Table 1. MGAS2221 is a fully sequenced *emm1* strain that is representative of the predominant global M1T1 lineage [69]. GAS strains were grown without agitation in Todd-Hewitt broth supplemented with 0.2% yeast (THY medium) at 37°C under 5% CO_2_. *Escherichia coli* strain DH5α was used for cloning and was grown at 37°C under agitation in Luria-Bertani (LB) medium supplemented with the respective antibiotic. Antibiotics were added at the following concentrations: ampicillin at 100 μg/ml, spectinomycin at 150μg/ml, and chloramphenicol at 25μg/ml.

### CovR antibody

CovR N-terminal domain (ND, amino acids 1–121) was amplified from GAS genomic DNA using primers CovR_ND__fwd and CovR_ND__rev (Table 1) and cloned into overexpression vector pET15b via *NdeI* and *XhoI* to generate pET15b-CovR_ND_. The recombinant protein was overexpressed in Rosetta (DE3)/pLysS (Novagen) by autoinduction [70], purified by gravity flow over TALON metal affinity resin (Clontech Laboratories) and eluted with 50 mM imidazole. Subsequently, the purified protein was concentrated in 20 mM Tris/HCl, 100 mM NaCl. Polyclonal antibody against purified CovR_ND_ protein was raised in rabbits by Covance Inc., Denver, and the serum was purified by affinity purification.

### Chromatin immunoprecipitation (ChIP)

GAS strains were grown in 40 ml THY medium to mid-exponential phase (OD~0.45). Proteins were cross-linked to DNA by adding formaldehyde to a final concentration of 1% and incubating for 10 minutes at room temperature. Subsequently, crosslinking was quenched with glycine at a final concentration of 0.125M, and cells were harvested at 4000 rpm for 10 minutes at 4° C. The cell pellets were washed twice with 20 ml of ice-cold phosphate-buffered saline (PBS) containing 1x cOmplete, EDTA-free protease inhibitor cocktail (Roche) and were then flash-frozen and stored at -80° C. The fixed cell pellets were resuspended in 1ml ice cold lysis buffer containing 50 mM Tris/HCl, pH 8.0, 150 mM NaCl, 1mM EDTA, 1% Triton X-100, 0.1% SDS, supplemented with cOmplete, EDTA-free protease inhibitor cocktail (Roche), incubated on ice for 10 minutes and transferred to three 1.5 ml Bioruptor tubes (Diagenode). Lysates were sonicated for 15 cycles (30s on/30s off) at 4° C in a Diagenode Bioruptor Plus machine set at high power, to shear DNA to fragments of 200 and 400 bp length. After centrifugation at 9000 rpm for five minutes, the supernatant was collected to use for chromatin-immunoprecipitation (950μl) or input DNA (50μl), respectively. CovR-bound DNA fragments were immunoprecipitated using a polyclonal antibody directed against the N-terminal domain of CovR (CovR_ND_). To this end, 75μl Dynabeads protein G (Invitrogen) in binding buffer (PBS buffer, 0.05% (vol/vol) Tween-20) was coated over night at 4°C with 15μg anti-CovR_ND_ antibody. The pre-coated beads were then incubated under rotation over night at 4°C with sonicated cell lysate. After removing the supernatant, the antigen-antibody-bead complexes were washed six times with 600μl lysis buffer, twice with RIPA500 buffer (10mM Tris/HCl, pH 8.0, 1mM EDTA, 500mM NaCl, 1% Triton-X-100, 0.2% SDS, and 0.1% DOC), twice with LiCl buffer (10mM Tris/HCl, pH 8.0, 1mM EDTA, 250mM LiCl, 0.5% (vol/vol) NP-40, and 0.5% (wt/vol) Na-DOC), followed by TE-buffer. After air-drying the beads for five minutes, the complex was incubated for five minutes at room temperature with 50μl elution buffer containing 10 mM Tris/HCl, pH 8.0, 5 mM EDTA, 300 mM NaCl, and 0.5% SDS to obtain the ChIP DNA (output) samples. The proteins and RNA in both input and ChIP samples were degraded by adding 100μg RNaseA and 200μg proteinase K and incubating for 2 h at 37° C and another 2 h at 55°C. Subsequently, crosslinking was reversed by incubation at 65° C overnight. The supernatant containing de-crosslinked ChIP or input DNA, respectively, was then purified using 150μl SPRI beads (AMPure XP, Beckman Coulter) on a magnetic stand. After washing twice with 80% ice-cold ethanol, the DNA was eluted with 10mM Tris/HCl, pH 8.0. The concentration of ChIP DNA and input DNA was determined on a Qubit machine 4.0 (Invitrogen) following the Qubit manual for high sensitive DNA. Generally, 40–80 ml cells grown to mid-exponential phase yielded ~10 ng ChIP DNA. DNA fragment size distribution was assessed using Agilent D1000 Screen Tape on an Agilent 2200 TapeStation system and had an ideal length of 150–300 bp. Four biological replicates per strain were used for sequencing.

### Generation of ChIP-seq data

ChIP sequencing was performed in the Advanced Technology Genomics Core (ATGC) Facility at MD Anderson Cancer Center. Illumina compatible indexed libraries were prepared from 2-10ng of sheared ChIP or input DNA using the KAPA Hyper Library Preparation Kit (KAPA Biosystems, Inc.). Libraries were amplified by 9 cycles of PCR, then assessed for size distribution using the 4200 TapeStation High Sensitivity D1000 ScreenTape (Agilent Technologies) and quantified using the Qubit dsDNA HS Assay Kit (Thermo Fisher). Equimolar quantities of the indexed libraries were multiplexed with 8 libraries per pool. The pool was quantified by qPCR using the KAPA Library Quantification Kit (KAPA Biosystems), then sequenced on an Illumina NextSeq500 sequencer using the high-output 75nt single read flow cell format.

### Analysis of ChIP-seq data

Raw sequencing reads (~30-35M reads per replicate/input sample) were quality filtered, trimmed, and mapped to the reference genome MGAS2221 (NZ_CP043530.1) using CLC Genomics Workbench (v 21, Qiagen). Peaks representing potential CovR binding were identified using the Transcription Factor ChIP-seq module of CLC Genomics Workbench. Briefly, following peak shape learning and filtering by the CLC ChIP-seq analysis tool, peaks are assigned a score and *P*-value. Subsequently, reads mapping to individual peaks were enumerated and normalized [(total reads mapped to peak)/ (peak length in bp) *1000; reads per kilobase length–RPKL). Peak shape scores and RPKL values were plotted against each other and peaks manually inspected to identify a reliable threshold for statistically significant enrichment of DNA. Peaks meeting the criteria of peak shape score >30 and RPKL >100 in at least three of the samples were called as statistically significant and investigated further. These criteria were intentionally strict, and additional sites not meeting these criteria may still have biological implications on gene regulation. A gene was defined as being associated with an enriched DNA region if the peak center was within 300 bps of the promoter or the open reading frame of the gene. A DNA enriched region was specified as being located in a promoter when the peak center was within 300 bps of a transcriptional start site as defined by [32].

### Correlation of CovR binding and influence on gene expression

Binding data derived from ChIP sequencing was correlated to previously generated transcriptome data (RNAseq) of the *emm1*-type strain MGAS2221 [29,30]. The CovR regulon was defined as gene with ≥ 2-fold difference in transcript levels between MGAS2221 and 2221Δ*covR* or between CovR strains with different CovR~P levels (M1-CovR-D53A vs. M1-CovS-T284A) [30] and based on previous biochemical and genetic studies [18,20,21].

### Motif search

A search for a CovR consensus motif in the regions bound by CovR *in vivo* was conducted using Multiple Em for Motif Elicitation (MEME), suite 5.1.1 [53,54]. The 45 input sequences employed in this search were 400bp in length (200bp +/- average peak center) and included both promoter and coding regions. Sequences with low RPKL and lacking influence on regulation based on previously determined CovRS transcriptome data [30] were excluded from the search. Over 50 MEME runs were performed with different combinations of program parameters. The most reliable motif based on biological background and E-value was identified with the motif discovery algorithm run in classic mode with site distribution allowing one occurrence per sequence (oops) and no palindromes and when the program was set to find up to three motifs with a width of 18–20 bp. An E-value of <0.05 was required for statistical significance of the motif [53]. Logo sequences were created using WebLogo [71].

### SYBR quantitative real-time polymerase chain reaction (qRT-PCR)

Enrichment of selected promoters in the ChIP samples derived from strain MGAS2221, M1-CovS-E281A, and 2221Δ*covR* relative to input DNA (cell lysate before ChIP) was assessed by SYBR qRT-PCR on a StepOne Plus machine (Applied Biosystems) using Ssoadvanced Universal SYBR Green Supermix (Bio-Rad) and the primers listed in S1 Table. As internal control, fold enrichment of promoters under investigation was normalized to enrichment of the *ldh* promoter region, a promoter that is not regulated by CovR. Measurements were done in duplicate on at least two biological samples.

### TaqMan qRT-PCR analysis

Strains were grown in THY media to mid-exponential phase and RNA was isolated using the RNAeasy Minikit (Qiagen). Approximately 300 ng RNA per sample was converted to cDNA using a high-capacity reverse transcription kit (Applied Biosystems). TaqMan qRT-PCR was performed on an Applied Biosystems Step-One Plus system using primers and probes listed in S1 Table. At least two biological replicates were analysed on two separate occations (n = 4). Transcript levels between different strains were compared using a two-sample *t* test (unequal variance) with a P value of ≤ 0.5 following Bonferroni’s adjustment for multiple comparison and a mean transcript level of ≥ 2-fold change being considered as statistically significant different.

### Luminescence assay

A ~200 bp long promoter fragment of *hasA* and *spyM1_0187*, amplified from MGAS2221 genomic DNA using primers listed in S1 Table, was cloned via *EcoRI* and *NotI* to pJC306 (kindly provided by M. Federle). Mutations within the putative CovR binding motif were introduced by quick-change mutagenesis (see S1 Table for primers). The plasmid harboring the wild type or mutated promoter-*luxAB* reportergene fusion was transformed into GAS strains MGAS2221 and 2221Δ*covR*, respectively. Cells were grown in THY, and luminescence was measured in a Biotek Synergy2 plate reader as previously described [72]. The experiments were performed at least twice using three independent biological replicates. Luminescence activity was determined in relation to OD_600_ and reported in relative light units (RLUs).

## Supporting information

S1 Fig Genomic context and CovR binding in the (*A*) *sag* operon, (*B*) fibronectin-collage-T antigen (FCT) region including the hypothetical protein encoding gene *MGAS2221_0159*, (*C*) *dpp* operon, (*D*) *nga/slo* operon region including the hypothetical genes *MGAS2221_0187/8*, and *(E)* the *speB* operon region including *grm*. Genes are represented by solid, horizontal arrows in the direction of transcription, and relevant genes are labeled on top. Below, depth of mapped reads generated by ChIP-seq of strains MGAS2221 and 2221-CovS-E281A, respectively, are shown in blue with peaks indicating CovR binding sites. Y-axis indicates 1000x depth of read mapping.
(EPS)

S2 Fig CovR does not influence transcript levels of *codY*. TaqMan qRT-PCR analysis showing *in vivo* transcript levels of *codY* in strains MGAS2221 (wild type, blue) and 2221Δ*covR* (red) grown to mid-exponential phase in THY medium. Data graphed are means ± standard deviation derived from four independent biological replicates measured in duplicate.
(AI) Click here for additional data file.

S3 Fig DNA binding in the OmpR/PhoB family of transcriptional regulators. Shown are DNA binding sequences for selected OmpR/PhoB family proteins for which structures of protein-DNA complexes are available (PDB entry in parentheses). Black lines indicate the tandem binding motifs, respectively, and the spacer length between these motifs is given. Bases involved in specific base contacts with the respective protein are labeled in green.
(EPS)

S4 Fig Genomic context and CovR binding in the (*A*) *covRS* operon region, (*B*) *codY* region, (*C*) *has* operon region. Genes are represented by solid, horizontal arrows in the direction of transcription, and relevant genes are labeled on top. Below, depth of mapped reads generated by ChIP-seq of strains MGAS2221 and 2221-CovS-E281A, respectively, are shown in blue with peaks indicating CovR binding sites. Y-axis indicates 1000x depth of read mapping.
(EPS)

S1 Table Primers and probes. Primers and probes used in this study.
(DOCX)

S1 Data Numerical data. Numerical data used in all figures.
(XLSX)
