# Supplementary material for: CovS inactivation reduces CovR promoter binding at diverse virulence factor encoding genes in group A Streptococcus
Source: PLoS Pathog. 2022 Feb 18;18(2):e1010341. doi: 10.1371/journal.ppat.1010341 (PMC8893699; doi:10.1371/journal.ppat.1010341)
Supplement: S1 Table — Primers and probes used in this study. (DOCX) [file ppat.1010341.s005.docx]

**S1 Table Primers and probes**

| **Name** | **Sequence** | **Description** |
| --- | --- | --- |
| CovR_ND__fwd | ATAATAATACATATGACAAAGAAAATTTTAATTATTG | cloning, overexpression |
| CovR_ND__fwd | ATAATAATAGGATCCTTAATCTTGACGGCGGAAAATAG | cloning, overexpression |
| hasA_reporter_fwd | ATAATAATAGAATTCGACTTAATTGTC GGGATTCT | cloning, reporter-gene fusion |
| hasA_reporter_rev | ATAATAATAGCGGCCGCCCTCCTACTTTTTTAATTTCCTTGAAAG | cloning, reporter-gene fusion |
| spy_0187_rep_fwd | ATAATAATAGAATTCAAAGCTGTAACACCTCA | cloning, reporter-gene fusion |
| spy_0187_rep_fwd | ATAATAATAGCGGCCGCCTCCTTAAATTATAGTATACC | cloning, reporter-gene fusion |
| has_mut2,5_fwd | CTGTCTTTCAAGGAACTTCAAAAAGTAGGAGGG | quick-change mutagenesis |
| has_mut2.5_rev | CCCTCCTACTTTTTGAAGTTCCTTGAAAGACAG | quick-change mutagenesis |
| has_mut3,4_fwd | CTGTCTTTCAAGGAAACCAAAAAAGTAGGAGGG | quick-change mutagenesis |
| has_mut3,4_rev | CCCTCCTACTTTTTTGGTTTCCTTGAAAGACAG | quick-change mutagenesis |
| spy_0187_mut10,13 fwd | TCGGTCAAACACATAACAAACAGGTTAAAATGTGATATTG | quick-change mutagenesis |
| spy_0187_mut10,13_ rev | CAATATCACATTTTAACCTGTTTGTTATGTGTTTGACCGA | quick-change mutagenesis |
| spy_0187_mut2,5 fwd | TCGGTCAAACACCTACCAAAAAGATTAAAATGTGATATTG | quick-change mutagenesis |
| spy_0187_mut2,5 rev | CAATATCACATTTTAATCTTTTTGGTAGGTGTTTGACCGA | quick-change mutagenesis |
| spy_0187_mut3,4 fwd | TCGGTCAAACACACCACAAAAAGATTAAAATGTGATATTG | quick-change mutagenesis |
| spy_0187_mut3,4 rev | CAATATCACATTTTAATCTTTTTGTGGTGTGTTTGACCGA | quick-change mutagenesis |
| spy_0187_mut14,15 fwd | TCGGTCAAACACATAACAAAAAGACCAAAATGTGATATTG | quick-change mutagenesis |
| spy_0187_ mut14,15 rev | CAATATCACATTTTGGTCTTTTTGTTATGTGTTTGACCGA | quick-change mutagenesis |
| spy_0187_sp+2_fwd | TCGGTCAAACACATAACAGCAAAAGATTAAAATGTGATATTG | quick-change mutagenesis |
| spy_0187_sp+2_rev | CAATATCACATTTTAATCTTTTGCTGTTATGTGTTTGACCGA | quick-change mutagenesis |
| spy_0187_sp+4_fwd | TCGGTCAAACACATAACA**GC**GCAAAAGATTAAAATGTGATATTG | quick-change mutagenesis |
| spy_0187_sp+4_rev | CAATATCACATTTTAATCTTTTGCGCTGTTATGTGTTTGACCGA | quick-change mutagenesis |
| ahpC_SYBR_fwd | CATAATATTAAGTTGCCTTAACGGA | SYBR qRT-PCR |
| ahpC_SYBR_rev | TCTGACAATTAAGTTGTGAAATAAATC | SYBR qRT-PCR |
| ska_SYBR_fwd | AT TAT CAT GAC ATT ATC ATT AAG | SYBR qRT-PCR |
| ska_SYBR_rev | AGA AAC CTC CTA AAA GTT AAG | SYBR qRT-PCR |
| covR_SYBR_fwd | CTTGCAAGGGTTGTTTGATG | SYBR qRT-PCR |
| covR_SYBR_rev | GAAGCATCTAATAGTCATTACCTTC | SYBR qRT-PCR |
| 0159_SYBR_fwd | GCT AGT AGT GAA GTG AAG AG | SYBR qRT-PCR |
| 0159_SYBR_rev | CGC TTC ACT CAT CAA CAA TG | SYBR qRT-PCR |
| prtS_SYBR_fwd | GTT ACA AGG CTT TCG TTT AAC | SYBR qRT-PCR |
| prtS_SYBR_rev | CCT GAT ACC CTC CTA AAT GT | SYBR qRT-PCR |
| codY_SYBR_fwd | AGATCCAGATCACTTCCGTAT | SYBR qRT-PCR |
| codY_SYBR_rev | GTTAGAGCATAGGGTCACATTC | SYBR qRT-PCR |
| braB_SYBR_fwd | TGTGCTAAGAATTTTCAGTCAAT | SYBR qRT-PCR |
| braB_SYBR_rev | CATGAATCCAATGACGATATATACA | SYBR qRT-PCR |
| sic_SYBR_fwd | AAGCTGACCTTTACTAATAATCGTC | SYBR qRT-PCR |
| sic_SYBR_rev | CACGGCTACAAGGGATGT | SYBR qRT-PCR |
| speB_SYBR_fwd | TGTCCATTAGTTGACTCGTAGG | SYBR qRT-PCR |
| speB_SYBR_rev | GAGAAGTCAATAAAAGCATTGACA | SYBR qRT-PCR |
| sagA_SYBR_fwd | CTAGAGTTATCAAAATGATATAAG | SYBR qRT-PCR |
| sagA_SYBR_rev | CAACTATCTAGTTCTTATCAC | SYBR qRT-PCR |
| grab_SYBR_fwd | ACTTGATTTTATGCAATTAATCTGT | SYBR qRT-PCR |
| grab_SYBR_rev | ACTAATCCAAAAGCTGATCTACG | SYBR qRT-PCR |
| 188_SYBR_fwd | GGAATTTAAATAACCATCTCGTG | SYBR qRT-PCR |
| 188_SYBR_rev | AGCAGTTGCGATCATAAATAAAG | SYBR qRT-PCR |
| sdaD2_SYBR_fwd | GACAAACTAACTTATTAACTATTGACGAA | SYBR qRT-PCR |
| sdaD2_SYBR_rev | AGTGAATAATTAGATGTCTCCAATGT | SYBR qRT-PCR |
| cfa_SYBR_fwd | CAGACAAAGTCAGCTAGACGAT | SYBR qRT-PCR |
| cfa_SYBR_fwd | TTAATGTCATTAAATGCCTAGTATTACT | SYBR qRT-PCR |
| codY 5’ | GAC TAT TGC GCC GAT TTA TGG | TaqMan qRT-PCR |
| codY 3’ | CCA AAA TCA AAT CAT CAT CAC TAA ACT C | TaqMan qRT-PCR |
| codY probe | ATG CGC CTT GGA TCA CTC ATT ATC TGG C | TaqMan qRT-PCR |
